# Chitosan-based edible coating produced from an emulsion containing the major constituents of oregano essential oil for the control of sour rot in ‘Lima’ orange

**DOI:** 10.1007/s42770-026-02125-1

**Published:** 2026-10-23

**Authors:** Adriane Maria da Silva, Daniel Terao, Itala Suzana Oliveira Silva, Marcos Augusto Bizeto, Fernanda Ferraz Camilo, Sonia Claudia do Nascimento de Queiroz, Aline de Holanda Nunes Maia, Elke Simoni Dias Vilela, Aldemir Chaim, Juliana Aparecida Fracarolli

**Affiliations:** 1 Faculty of Agricultural Engineering (FEAGRI), State University of Campinas, Postharvest Technology Laboratory, Cidade Universitária Zeferino Vaz, Barão Geraldo, 13083-970 Campinas, SP Brazil; 2 Embrapa Meio Ambiente, Empresa Brasileira de Pesquisa Agropecuária, CP 69, 13918-110 Jaguariúna, SP Brazil; 3 Graduate Program in Food Science, Federal University of Bahia (UFBA), 40170-110 Salvador, BA Brazil; 4 Institute of Chemical, Environmental, and Pharmaceutical Sciences (ICAQF), Department of Chemistry, Federal University of São Paulo (UNIFESP), 09913-030 Diadema, SP Brazil

**Keywords:** Chitosan emulsion, Oregano essential oil, *Geotrichum citri-aurantii*, Citrus, Quality attributes, Defense responses

## Abstract

Sour rot caused by *Geotrichum citri-aurantii* is one of the most destructive postharvest diseases affecting citrus fruits, mainly the ‘Lima’ variety, leading to severe economic losses. Beyond its economic impact, the management of postharvest diseases is linked to global efforts to reduce food losses and decrease reliance on synthetic agrochemicals, aligning with the Sustainable Development Goals related to responsible production. In this context, active coating-based strategies have emerged as promising alternatives to conventional fungicides. In this study, in vitro and in vivo experiments were conducted to evaluate the antifungal capabilities of an emulsion produced by mixing a chitosan solution with the major constituents of oregano (*Origanum vulgare)* essential oil - carvacrol, thymol, and *p-cymene*. We also treated postharvest oranges with this emulsion to evaluate. The physicochemical quality and defense enzyme activity. The formulated emulsion exhibited a broad oil droplet size distribution in the micrometric range and a good film-forming capacity. In vitro experiments demonstrated complete inhibition of colony formation at 0.25 µL mL⁻¹ and inhibition of mycelial growth at 3.00 µL mL⁻¹, whereas lower antifungal efficacy was observed in the vapor phase. When applied to orange fruit, the emulsion at 7.5 µL mL⁻¹ reduced disease severity. The treatment also maintained higher firmness values and more stable pH and color attributes at the evaluated sampling times. Changes in the activity of enzymes were also observed at specific sampling times following treatment. Overall, the results indicate that the emulsion is a promising alternative for controlling sour rot while preserving the postharvest quality of citrus fruits.

**Supplementary Information:**

The online version contains supplementary material available at https://doi.org/10.1007/s42770-026-02125-1.

## Introduction

Citrus production is one of the most economically significant agricultural activities worldwide [1], however, postharvest losses caused by fungal diseases severely compromise the profitability of the sector. Among the most impactful pathogens, *Geotrichum citri-aurantii*, the causal agent of sour rot, stands out due to its aggressiveness and rapid spread in citrus fruits [2]. Unlike *Penicillium digitatum* and *Penicillium italicum* - traditionally the primary targets of postharvest control - *G. citri-aurantii* exhibits greater capacity for tissue penetration and proliferation, in addition to reduced sensitivity to conventional fungicides, making it one of the most challenging pathogens to manage [3]. This disease affects several citrus species and cultivars, among them, the ‘Lima’ variety is particularly relevant due to its importance in the fresh fruit market and its susceptibility to sour rot [4], making it a suitable model for evaluating alternative postharvest control strategies in citrus.

Currently, sour rot management is primarily based on synthetic fungicides such as imazalil and thiabendazole [3, 5]. However, prolonged use of these molecules has led to the selection of resistant strains and accumulation of undesirable chemical residues, posing potential risks to human health and the environment [5]. In this context, postharvest alternatives based on coatings have received increasing attention [6].

Coating with essential oils has been investigated due to its broad antimicrobial activity [7, 8]. However, their practical application is often limited by high production costs, chemical variability, extraction conditions, strong aroma, and instability under storage conditions [9]. To overcome these limitations, the use of major constituents of essential oils with recognized antifungal activity has emerged as a more standardized and economically viable strategy [9]. Carvacrol, thymol, and p-cymene, which are major constituents of oregano essential oil (*Origanum vulgare*), exhibit antifungal effects, primarily through disruption of fungal cell membranes [10, 11]. Importantly, carvacrol and thymol, as well as the terpene *p-cymene*, are included in regulatory inventories of food additives and flavoring agents maintained by the U.S. Food and Drug Administration [12–14]. In addition, chitosan has been the subject of GRAS notifications evaluated by the U.S. Food and Drug Administration [15] supporting its use in food-related applications. These regulatory considerations reinforce the potential of these compounds as safe alternatives to synthetic fungicides for postharvest treatment of fresh fruit.

In a previous study, the combination of these three chemical constituents demonstrated synergistic inhibitory activity against *G. citri-aurantii in vitro* [16]. Nevertheless, although their antifungal synergy has been demonstrated under controlled laboratory conditions, the practical application of these compounds on fruit surfaces is a challenge, as direct application may result in poor dispersion, rapid volatilization, and limited retention on the fruit surface [17].

In this context, incorporation into a polymeric matrix such as chitosan represents a promising strategy to improve stability, dispersion, and controlled release of these bioactive compounds. Chitosan is a semi-synthetic biopolymer derived from chitin, the second most abundant biopolymer after cellulose, and has been widely explored for edible coating applications due to its film-forming ability, and biodegradability [18, 19]. When applied as a coating, chitosan films adhere to fruit tissues, reduce water loss, and serve as carriers for bioactive compounds [18–21].

Furthermore, chitosan plays an important role as a natural emulsifying and stabilizing agent. Its chemical structure contains hydrophobic acetylated groups and hydrophilic deacetylated amino groups, enabling electrostatic and steric stabilization of oil droplets in emulsion, thus preventing coalescence [22]. The formation of a stable chitosan-based emulsion may therefore represent a viable technological approach to “package” synergistic antifungal compounds into a formulation suitable for practical postharvest application.

In addition to direct antifungal effects, bioactive compounds with chitosan may stimulate host defense mechanisms in fruits [23]. Therefore, evaluating enzymatic responses is essential to determine whether the proposed formulation acts not only through direct antifungal activity, but also through the fruit’s natural defense mechanisms.

Given this context, this study aimed to evaluate the antifungal activity of a stable chitosan-based emulsion containing the major constituents of oregano essential oil - carvacrol, thymol, and *p-cymene*, on the in vitro control of *G. citri-aurantii* and on sour rot in orange fruit variety ‘Lima’, as well as to investigate its effects on physicochemical quality attributes and on the induction of defense-related enzyme activities.

## Materials and methods

### Fungal isolate

*Geotrichum citri-aurantii*, isolated from an orange variety ‘Lima’, was obtained from the Collection of Microorganisms of Agricultural and Environmental Importance (CMAA, [24], 2025) at Embrapa Meio Ambiente, Jaguariúna, São Paulo, Brazil. The isolate was cultured on potato dextrose agar (PDA) and maintained in a controlled environment chamber at 22 ± 2 °C, 70 ± 2% relative humidity, for 7 days before the experiments.

### Emulsion preparation

The emulsion, containing the major components of oregano essential oil and acidic aqueous chitosan solution, was prepared as follows. A 5% (v/v) emulsion was prepared by mixing 5 mL of a synthetic mixture containing the three major constituents of oregano essential oil, carvacrol, p-cymene, and thymol (Sigma-Aldrich, St. Louis, Missouri, USA), at mass fractions of 77.2% carvacrol, 18.5% p-cymene, and 4.3% thymol, with 95 mL of a 2% (w/v) chitosan solution using an ultrasonic processor (Sonic VCX 130). This resulted in a stock emulsion equivalent to 50 µL mL⁻¹. The densities, volumes, masses, and corresponding mass fractions of the individual constituents used to prepare the synthetic mixture are presented in Supplementary Table S1.

The 2% (w/v) chitosan solution was prepared by dissolving the appropriate amount of chitosan (Sigma-Aldrich; 80% degree of deacetylation) in a 2% (v/v) aqueous acetic acid solution under continuous mechanical stirring at room temperature.

The resulting emulsion was characterized by dynamic light scattering (DLS) and electrophoretic mobility measurements using a Zetasizer Advance Pro (Malvern Panalytical Ltd.). The emulsion was polydisperse (polydispersity index = 1.0), with oil droplets in the micrometer range and a Z-average hydrodynamic diameter of 7344 nm (7.344 μm), and a zeta potential of + 17.64 mV. The microstructural organization of the oil droplets was also examined by confocal laser scanning microscopy using a Leica TCS SP8 AOBS microscope. Representative micrographs and the main physicochemical parameters are presented in Supplementary Figure S1.

The 5% (v/v) stock emulsion was subsequently diluted with the same 2% (w/v) acidic chitosan solution to obtain the concentrations required for the in vitro and in vivo antifungal experiments, as well as for the enzymatic and physicochemical analyses performed on postharvest oranges.

### Evaluation of in vitro antifungal activity

The antifungal activity of the emulsion was evaluated using three methods. These experiments were designed to distinguish the inhibitory effects caused by volatile compounds from those resulting from direct contact between the emulsion and the pathogen.

#### (I) Indirect inhibition (volatile phase)

An aliquot of 414 µL of the chitosan-based emulsion containing the synthetic mixture at final concentrations of 30, 40, and 50 µL mL⁻¹ were dispensed over the inner surface of the lids of 90-mm Petri plates. These concentrations were selected based on preliminary experiments to characterize the concentration-dependent antifungal response under volatile-phase exposure. The formulations were spread evenly using a sterile Drigalski spatula and allowed to dry for 5 min under laminar airflow. The maximum concentration evaluated in this experiment (50 µL mL⁻¹) was limited by the physicochemical stability of the formulation [25].

On the base of each plate, containing PDA, a sterile filter paper disk (6 mm) impregnated with 10 µL of *G. citri-aurantii* spore suspension (10⁵ spores mL⁻¹) was placed at the center [26]. Control plates received only the disk with suspension. After sealing, plates were incubated at 22 ± 1 °C and 70 ± 2% relative humidity, with daily assessments of mycelial growth using a digital caliper for 5 days.

The applied volume (414 µL per plate) was scaled according to the estimated surface area of the fruit and the Petri plate. Based on preliminary tests, 1 mL of formulation was sufficient to uniformly cover an orange fruit with an approximate diameter of 70 mm. Assuming an approximately spherical fruit, its estimated surface area was 153.94 cm², corresponding to an application rate of approximately 6.50 µL cm⁻² (1000 µL/153.94 cm²). For the 90-mm Petri plate, with an internal surface area of 63.62 cm², the equivalent application volume was 414 µL per plate (6.50 µL cm⁻² × 63.62 cm²). This scaling was used to standardize the formulation volume according to the exposed surface area.

#### (II) Direct inhibition by deposition of the emulsion over the spore suspension

A volume of 100 µL of *G. citri-aurantii* spore suspension (10³ spores mL⁻¹) was uniformly spread over the PDA surface using a Drigalski spatula and allowed to dry for 5 min under laminar flow. Subsequently, 414 µL of the chitosan-based emulsion containing the synthetic mixture at final concentrations of 0.10, 0.25, and 0.50 µL mL⁻¹, were spread on the surface [27]. These concentrations were selected based on preliminary experiments and were lower than those used in the other direct-contact assay because the emulsion was applied directly onto the spore suspension, resulting in immediate contact between the formulation and the spores. Control plates received only the spore suspension. Plates were sealed and incubated at 22 ± 1 °C and 70 ± 2% relative humidity for 3 days. After the third day of incubation, colony-forming units (CFU) were counted. A spore suspension of 10³ spores mL⁻¹ was used in this experiment to obtain approximately 100 colonies per plate, facilitating CFU counting.

#### (III) Direct inhibition by deposition of the spore-containing disk over the emulsion

A volume of 414 µL of the chitosan-based emulsion containing the synthetic mixture at final concentrations of 2, 3, and 4 µL mL⁻¹ was applied over the PDA medium, spread uniformly, and allowed to dry under laminar flow for 5 min. At the center of each plate, a sterile filter paper disk (6 mm) impregnated with 10 µL of *G. citri-aurantii* spore suspension (10⁵ spores mL⁻¹) was placed [28]. Control plates received only the disk with suspension. After incubation at 22 ± 1 °C and 70 ± 2% relative humidity for 5 days, mycelial growth was assessed using a digital caliper.

All control plates were kept under the same storage conditions as the treated plates. All in vitro experiments were conducted in a completely randomized design, with four replicates (plates) per treatment. The percentage of growth inhibition was calculated relative to the control treatment, using mycelial growth (mm) measured on the final day of evaluation or CFU counts.

### Fruit preparation

Orange fruit variety ‘Lima’ were obtained from commercial growers in the Campinas region, São Paulo, Brazil, at ripeness stage 3 according to the [29], characterized by entirely orange-colored peel. Fruit were washed with water and neutral detergent, rinsed under running water, and dried with paper towels, then stored at 20 ± 2 °C and 80 ± 2% relative humidity. For enzymatic analyses, fruit were washed only with running water, without detergent.

### Evaluation of in vivo antifungal activity

Orange fruit were wounded at the equatorial region using a stainless-steel needle (1 mm × 3 mm) and inoculated with 10 µL of the *G. citri-aurantii* suspension (10⁶ spores mL⁻¹). After inoculation, fruit were placed in plastic boxes with lids and kept in a humid chamber for 4 h at 22 ± 2 °C [4]. The sealed boxes were used to provide a high-humidity environment favorable for pathogen infection.

Subsequently, a chitosan-based emulsion containing the synthetic mixture at final concentrations of 2.5, 5.0, 7.5, and 10.0 µL mL⁻¹, was applied by spraying at a rate of 1 mL per fruit. These concentrations were selected to provide a range of increasing emulsion concentrations for evaluating disease control and potential effects on the fruit surface. Applications were performed using a pneumatic spray nozzle producing droplets of 10–30 μm, positioned approximately 5 cm from the fruit surface. Inoculated fruit receiving no treatment served as the control.

The experiment was conducted in a completely randomized design with 10 independent fruits per treatment, with each fruit considered one biological and experimental unit. Fruit was stored at 26 ± 2 °C and 90 ± 2% relative humidity in a controlled storage chamber. Temperature and relative humidity were monitored throughout the experimental period using the chamber’s built-in monitoring system. Disease development was monitored on the same individually identified fruits throughout the assessment period.

Disease incidence and severity were evaluated daily for six days after inoculation. Disease incidence was expressed as the percentage of symptomatic fruit, whereas disease severity was assessed by measuring lesion diameter on each fruit using a digital caliper. Disease progression was quantified by calculating the area under the disease progress curve (AUDPC) from lesion diameter values recorded throughout the six-day assessment period, using the trapezoidal method described by Campbell and Madden [30]. The incubation period was defined as the number of days from inoculation to the first visible appearance of symptoms.

### Evaluation of postharvest quality

Orange fruit were sprayed with the emulsion at 7.5 µL mL⁻¹, the concentration that showed the best control of sour rot in the in vivo experiment. The fruit was stored at 20 ± 2 °C and 80 ± 2% to simulate marketing conditions. Analyses were performed after 2, 6, and 8 days of storage, using 10 replicates (fruit) per treatment at each sampling time. The experiment followed a completely randomized design and two treatments were evaluated: an untreated control and fruit treated with the emulsion. The fruit was not artificially inoculated with the pathogen.

Mass loss was determined by weighing on an analytical balance, with results expressed as a percentage of the initial weight [31]. Peel color was measured using a CM-700d colorimeter (Konica Minolta) under D65 illuminant (6500 K) and 10° viewing angle, resulting in the L*, a*, and b* values. Chroma (C*) and hue angle (h) were calculated [32]. Firmness was measured with a texture analyzer (TA500, Lloyd Instruments), using a 490 N load cell, 75 mm plate probe, 5% deformation, and a speed of 1 mm s⁻¹ [33], with results expressed in Newtons (N).

Juice extracted using a manual squeezer and filtered through gauze was used to determine soluble solids (SS) in a digital refractometer (°Brix, MA871 – Milwaukee Instruments), titratable acidity (TA) by titration of 5 mL of juice diluted in 45 mL distilled water with 0.1 N NaOH (Labsynth, 99% purity), standardized with Potassium Biphthalate (Vetec, 99.5% purity) to pH 8.1, with results expressed as % citric acid (Eq. 312IV, [34]), SS/TA ratio, and pH (pH meter Cole-Parmer 2500).

### Evaluation of enzymatic response

Two treatments were evaluated: a control and fruit treated with the emulsion at 7.5 µL mL⁻¹. The fruit were stored at 20 ± 2 °C and 80 ± 2% relative humidity. The experimental design was a completely randomized design, with four replicates (fruits) per treatment at each sampling time. Peel samples were collected at 2 h after treatment, and daily up to the sixth day. The fruit was not artificially inoculated with the pathogen.

Peel fragments (1 g) were ground in liquid nitrogen and homogenized in 6 mL of 50 mM sodium phosphate buffer (pH 6.5) (Na₂HPO₄ + NaH₂PO₄·H₂O, Sigma-Aldrich) containing 1% polyvinylpyrrolidone (PVP10, Sigma-Aldrich) and 1 mM phenylmethylsulfonyl fluoride (Sigma-Aldrich). Extracts were centrifuged at 20,000 × g for 25 min at 4 °C, and the supernatant was stored at − 18 °C [35].

Protein concentration was determined according to Bradford [36], using bovine serum albumin (BSA) as the calibration standard and Bradford reagent (B6916, Sigma-Aldrich). BSA standards covering a nominal concentration range of 0–1400 µg mL⁻¹ were analyzed under the same assay conditions as the samples by mixing 15 µL of standard solution or enzyme extract with 200 µL of Bradford reagent. Absorbance was measured at 595 nm between 2 and 10 min after reagent addition. Linear regression yielded the equation y = 0.0005097x + 0.39497 (R² = 0.98096). Although the calibration curve showed a positive linear trend, the coefficient of determination alone does not establish that the entire concentration range falls within the linear response range of the assay. Furthermore, the linear range was not independently validated for the specific assay conditions used in this study. Therefore, the nominal concentration range of the standards should not be interpreted as a fully validated linear range. Sample protein concentrations were calculated from the calibration curve and expressed as mg protein g⁻¹ fresh tissue.

Peroxidase activity was determined from the reaction containing 50 µL enzyme extract, 72 µL of 10 mM sodium phosphate buffer (pH 6.5), 72 µL of 3 mM hydrogen peroxide (H₂O₂; 6% solution, Scharlau), and 72 µL of 15 mM guaiacol (Sigma-Aldrich), monitored at 470 nm at 30 °C over three cycles of 20 s [37]. One enzyme unit (U) was defined as an absorbance change of 0.01 min⁻¹, and activity was expressed as U mg⁻¹ protein.

Polyphenoloxidase activity was measured using 20 µL of extract and 200 µL of 20 mM pyrocatechol (Exodo Científica) in 100 mM phosphate buffer (pH 6.8), with readings at 420 nm over four 30-second cycles at 30 °C [38]. Activity was expressed as U mg⁻¹ protein, where 1 U corresponded to an absorbance change of 0.01 min⁻¹.

For both peroxidase and polyphenoloxidase assays, reaction blanks without enzyme extract were included to account for background absorbance from the reaction components. The absorbance of the corresponding blank was subtracted from the absorbance of each sample before enzyme activity was calculated. The change in absorbance per minute (ΔAbs min⁻¹) was calculated as the slope of the absorbance-versus-time curve by linear regression using all readings obtained during the measurement.

Catalase activity was quantified by reacting 140 µL of extract with 70 µL of 10 mM hydrogen peroxide in 50 mM phosphate buffer (pH 7.0) [39], with absorbance measured at 240 nm over five 5-second intervals at 25 °C. Activity was calculated using Lambert–Beer’s law (A = ε·b·c), using a molar extinction coefficient (ε) of 39.4 M⁻¹ cm⁻¹ for H₂O₂ and an optical path length (b) of 0.62 cm adopted from the referenced assay protocol [39]. This path length was not experimentally determined for the specific reaction volume (210 µL) and 96-well microplate geometry used in the present study. Therefore, the calculated absolute catalase activity values may be affected by a systematic bias associated with the assumed optical path length. Catalase activity was expressed as µmol H₂O₂ min⁻¹ mg⁻¹ protein.

Phenylalanine ammonia-lyase activity was determined using 20 µL of extract, 90 µL of 100 mM borate buffer (pH 8.8) (H₃BO₃ + K₂B₄O₇·4 H₂O, Sigma-Aldrich), and 90 µL of 12 mM L-phenylalanine (Exodo Científica), incubated at 37 °C for 20 min [40]. The reaction was stopped with 15 µL of 6 N HCl (37%, Scharlau), and the formation of trans-cinnamic acid (TCA) was quantified by standard curve at 290 nm, expressed as µg trans-cinnamic acid min⁻¹ mg⁻¹ protein. The amount of potassium tetraborate tetrahydrate used (K₂B₄O₇·4 H₂O) was calculated based on the boron content of the compound; the molar mass was divided by four to correspond to the amount equivalent to 1 mol of boron.

All analyses were performed in 96-well microplates (Greiner Bio-One), and readings were obtained on an Infinite 200 PRO UV-Vis spectrophotometer (Tecan) using Magellan 7.2 software.

### Statistical analysis

The statistical procedures were selected according to the type and distribution of each response variable and the assumptions required for the corresponding parametric analyses.

The influence of emulsion concentration on mycelial growth inhibition (MGI) in the volatile-phase in vitro experiment was examined using a nonlinear exponential regression model (Eq. 1). The model was fitted using PROC NLIN in SAS/STAT^®^ [41] to describe the observed trend in MGI across the three tested emulsion concentrations (30, 40, and 50 µL mL⁻¹), with four replicates per concentration. Because only three concentration levels were evaluated, the regression was used as a descriptive representation of the observed response within the tested concentration range and was not intended to establish a robust concentration–response relationship or support extrapolation beyond this range.

1 $$\:{y}_{ij}={\beta\:}_{0}{e}^{{\beta\:}_{1{x}_{j}}}+\:{\epsilon\:}_{ij}$$

In regression model, *y*_*ij*_ represents the mycelial growth inhibition (MGI, %) observed at concentration *x*_*j*_ (30, 40 and 50 µL mL⁻¹) in replicate *i* (*i* = 1, 2, 3, 4); *β*_*0*_ is the model scale coefficient, representing the expected response when x = 0; *β*_*1*_ is the exponential rate coefficient describing the rate of change in MGI with increasing concentration; and *ε*_*ij*_ denotes the random error associated with each observation.

For the direct-contact in vitro experiments (emulsion over spores and spores over emulsion), the data were evaluated for normality and homogeneity of variances. Because these assumptions were not satisfied, nonparametric multiple-contrast procedures were used to compare emulsion concentrations within each application condition. The analyses were performed using the *nparcomp* package in R version 4.5.2 [42], with a user-defined contrast matrix (type = “*userdefined*”) and a one-sided alternative hypothesis (alternative = “*less*”). Treatments were considered significantly different at *p* < 0.05. The specific contrasts and their directions are provided in Supplementary Table S2 and S3. The two experimental methods were analyzed independently and were not statistically compared with each other because they involved different experimental procedures and response variables.

For the in vivo experiment, disease incidence was analyzed as a binary response (infected or non-infected fruit). Therefore, the incidence of each emulsion treatment was compared with that of the control using lower-tailed pairwise Fisher’s exact tests, performed with SAS PROC FREQ. A one-sided lower-tailed test was used because the a priori hypothesis was that the emulsion would reduce disease incidence. The significance level was set at *p* < 0.10 because this binary response was considered an exploratory outcome and the number of fruit evaluated per treatment was relatively limited.

Mean lesion diameter (MLD) at 6 days after inoculation and the area under the disease severity progress curve (AUDPC) were evaluated for normality and homogeneity of variances. Although homogeneity of variances was not rejected, the normality assumption was not satisfied because of the high frequency of zero values observed among treatments. Therefore, nonparametric Dunnett-type multiple contrasts were used to compare each emulsion treatment with the control. The analyses were performed using the *nparcomp* package in R, with a Dunnett contrast matrix (type = “*Dunnett*”) and a one-sided lower-tailed alternative hypothesis (alternative = “*less*”), reflecting the a priori expectation of lower disease severity and AUDPC in treated fruit. The specific contrasts and their directions are provided in Supplementary Table S4.

The incubation period was also analyzed using nonparametric Dunnett-type contrasts because the normality assumption was not satisfied. Each emulsion treatment was compared with the control using a one-sided upper-tailed alternative hypothesis (alternative = “*greater*”), reflecting the a priori expectation that the emulsion would delay symptom development and consequently increase the incubation period. Statistical significance was assessed at *p* < 0.05.

Overall, one-sided tests were used only when a directional a priori hypothesis could be defined based on the expected biological effect of the emulsion. Lower-tailed tests were therefore used for disease incidence, MLD, and AUDPC, for which a reduction relative to the untreated control was expected, whereas an upper-tailed test was used for the incubation period, for which an increase relative to the control was expected.

Physicochemical and enzymatic variables were analyzed separately at each sampling time using independent-samples t-tests [43] to compare the control and emulsion treatments. Student’s t-test was used when homogeneity of variances was not rejected by Levene’s test (*p* > 0.05), whereas Welch’s t-test was used when homogeneity of variances was rejected (*p* < 0.05). Statistical significance was assessed at α = 0.05 for each comparison. No adjustment for multiple comparisons across response variables or sampling times was applied because the analyses addressed treatment differences for specific outcomes at each sampling time. However, this approach does not control the family-wise error rate across all tests and increases the risk of Type I errors. Therefore, the results were interpreted with appropriate caution, considering the number of comparisons performed.

## Results

### In vitro antifungal activity evaluation

**Fig. 1:**
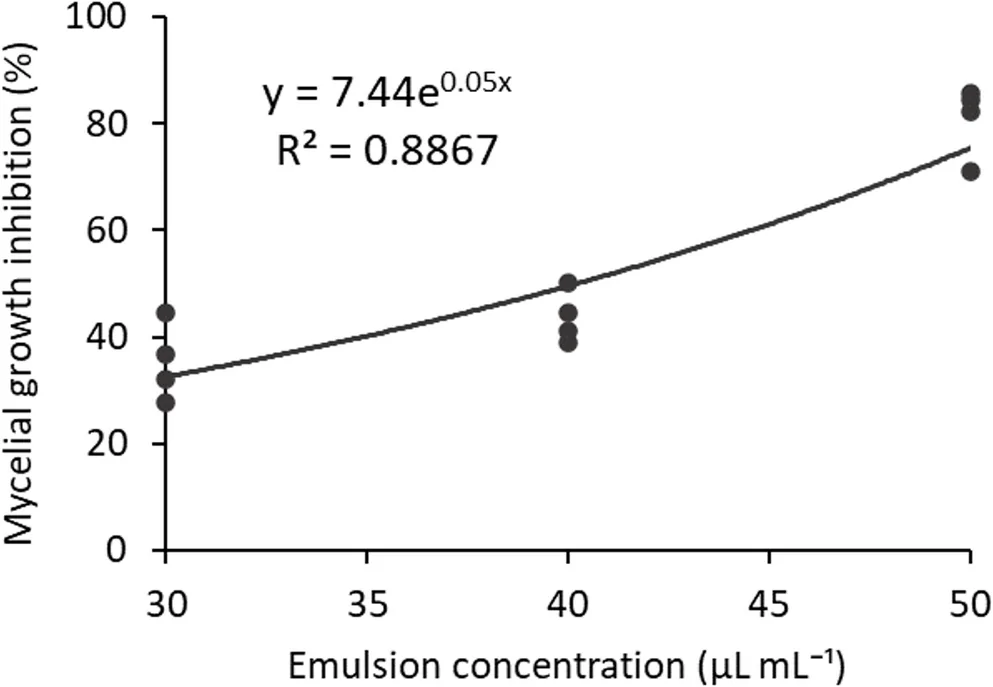
Nonlinear model fit to describe the relationship between the inhibition of *Geotrichum citri-aurantii* mycelial growth (y) in vitro and the concentration (x) of the emulsion applied in the volatile phase. Parameter estimates (standard error): b_0_ = 7.44 (1.99) and b_1_ = 0.05 (0.006); R² (determination coefficient) = 0.8867. The nominal significance levels (p-values) corresponding to each parameter were 0.0039 and < 0.0001, respectively

In the volatile-phase experiment (indirect-contact inhibition), MGI increased with increasing emulsion concentration and showed a progressively greater response at the highest concentration (Fig. 1). This concentration-response relationship was adequately described by the nonlinear exponential regression model. The estimated model coefficients were statistically significant (β₀ = 7.44 ± 1.99, *p* = 0.0039; β₁ = 0.05 ± 0.006, *p* < 0.0001), and the model explained 88.67% of the observed variability (R² = 0.8867). MGI increased gradually between 30 and 40 µL mL⁻¹ and more markedly at 50 µL mL⁻¹, indicating an increasingly pronounced inhibitory response at higher emulsion concentrations (Fig. 1; Figure S2).

In the direct contact experiment in which the emulsion was poured over the spore suspension, complete inhibition of spore germination was achieved at 0.25 µL mL⁻¹. In contrast, when the spore suspension was deposited over the emulsion-coated surface, a concentration of 3.0 µL mL⁻¹ was required to achieve complete inhibition of mycelial growth (Tables 1 and 2, Figure S2).

**Table 1:** Inhibition of *Geotrichum citri-aurantii* spore germination following application of the emulsion over spores under direct-contact in vitro conditions

Emulsion concentration (µL mL^− 1^)	Spore germination inhibition (%)
0.10	97.10 b ± 0.01
0.25	100.00 a ± 0.00
0.50	100.00 a ± 0.00

Results are presented as mean ± standard error (*n* = 4). Plates were incubated at 22 ± 1 °C and 70 ± 2% relative humidity for 5 days. Mean inhibitions followed by same letters do not differ by nonparametric contrasts (*p* < 0.05)

**Table 2:** Inhibition of *Geotrichum citri-aurantii* mycelial growth following application of spores over the emulsion under direct-contact in vitro conditions

Emulsion concentration (µL mL^− 1^)	Mycelial growth inhibition (%)
2.00	58.74 b ± 0.01
3.00	100.00 a ± 0.00
4.00	100.00 a ± 0.00

Results are presented as mean ± standard error (*n* = 4). Plates were incubated at 22 ± 1 °C and 70 ± 2% relative humidity for 5 days. Mean inhibitions followed by same letters do not differ by nonparametric contrasts (*p* < 0.05)

### Sour rot control

The application of the emulsion promoted a trend toward reduced incidence of sour rot in orange fruit inoculated with *G. citri-aurantii*, with the greatest numerical reduction observed at the concentration of 7.5 µL mL⁻¹, which resulted in 66.70% disease inhibition (Table 3).

**Fig. 2:**
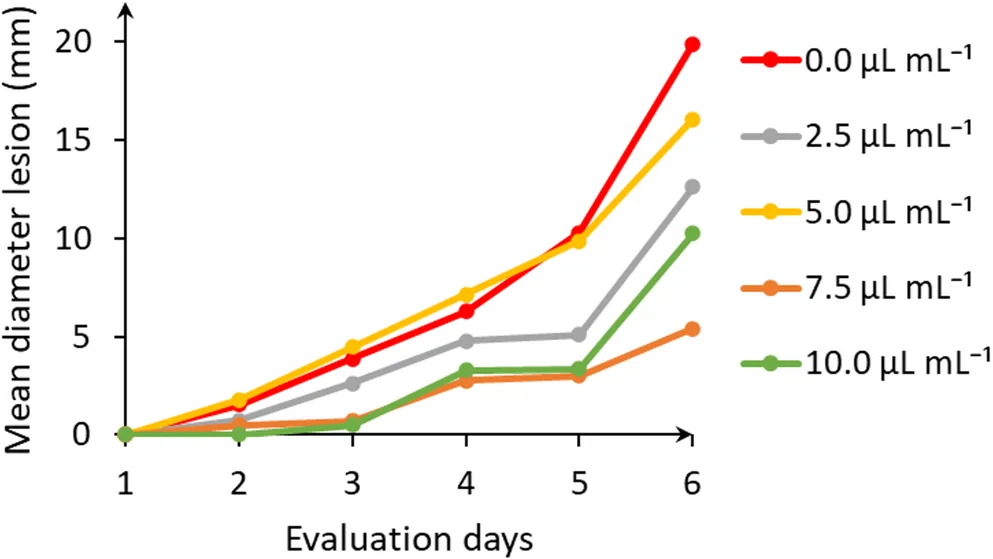
Sour rot progress curves in orange fruit treated with an emulsion containing chitosan and a mixture of carvacrol, thymol, *p-cymene*, — the major constituents of oregano essential oil — at concentrations of 0.0 (control), 2.5, 5.0, 7.5, and 10.0 µL mL⁻¹. The dots correspond to the estimated mean lesion diameter at each evaluation day (*n* = 10)

Regarding disease severity, untreated fruit (control) showed a rapid increase in lesion diameter, whereas fruit treated with 7.5 µL mL⁻¹ exhibited a slower disease progression (Fig. 2), as reflected by lower values of mean lesion diameter (MLD) and area under the disease severity progress curve (AUDPC) (Table 4). The 7.5 µL mL⁻¹ treatment also showed a numerically longer mean incubation period (5.20 days) than the control (4.30 days); however, this difference was not statistically significant.

It is noteworthy that the 10.0 µL mL⁻¹ treatment caused small superficial lesions on the peel surface. No visible epidermal damage was observed in the remaining treatments (Figure S3).

**Table 3:** Estimates of sour rot incidence, with corresponding standard errors (SE), and incidence inhibition in oranges treated with different emulsion concentrations, measured 6 days after inoculation

Emulsion concentration (µL mL^− 1^)	nº infected fruit	nº not infected	Incidence (%) ^(1)^	SE ^(2)^	Incidence inhibition (%)
0.0	6	4	60	15.50	-
2.5	4	6	40	15.50	33.30
5.0	3	7	30	14.50	50.00
7.5	2	8	20	12.60	66.70
10.0	3	7	30	14.50	50.00

^(1)^ Incidence values followed by * differ from the control (0.0 µL mL⁻¹) incidence by the lower-tailed Fisher’s exact test at the 0.10 significance level. ^(2)^ standard error of the estimate of the percentage of infected fruit (incidence)

**Table 4:** Estimated means per treatment (control and four emulsion concentrations) and respective standard errors (SE) for different response variables: sour rot severity in orange fruit, expressed by the mean lesion diameter (MLD) at day 6 after inoculation, area under the disease severity progress curve (AUDPC), and the disease incubation period (days)

Emulsion concentration (µL mL^− 1^)	MLD at 6 days (mm)		AUDPC		Incubation period (days)	
	Mean	SE ^(1)^	Mean	SE ^(1)^	Mean	SE ^(1)^
0.0	19.88	-	31.97	-	4.30	-
2.5	12.60 *	0.13	19.58 *	0.13	4.60	0.13
5.0	16.05 *	0.13	31.30 *	0.13	4.60	0.13
7.5	05.40 *	0.12	09.65 *	0.12	5.20	0.12
10.0	10.25 *	0.12	12.28 *	0.13	5.00	0.13

Asterisks (*) indicate significant differences between each emulsion treatment and the control (0.0 µL mL⁻¹) according to nonparametric Dunnett-type contrasts (*p* < 0.05). For MLD and AUDPC, lower-tailed contrasts were used because the a priori expectation was that the emulsion would reduce disease severity. For the incubation period, an upper-tailed contrast was used because the a priori expectation was that the emulsion would delay symptom development and consequently increase the incubation period. Homogeneity of variances was not rejected for MLD and AUDPC (Levene’s test, *p* > 0.05). However, the normality assumption was not satisfied because of the high frequency of zero values among treatments; therefore, nonparametric procedures were used

### Physicochemical analyses

The physicochemical parameters of oranges treated with the emulsion (7.5 µL mL⁻¹) and of the control during storage are presented in Table 5.

Fruit firmness showed lower values at later sampling times in both treatments; however, the coated oranges maintained higher values than the control up to the 8th day of storage. Weight loss showed progressively higher values at the successive sampling times; however, two days after treatment, emulsion-coated fruit exhibited significantly lower weight loss than control fruit.

The pH values showed a slight decrease at the successive sampling times, with higher values observed in the coated samples. Soluble solids showed higher values up to day 6, although a reduction was observed in the treated samples on day 8 compared with the control. Titratable acidity showed slightly lower values at later sampling times, while the SS/TA ratio showed progressively higher values across the sampling times, reaching higher values in the treated oranges at day 6.

Regarding color, increases in L* and a* values were observed in all fruit, with more pronounced numerical changes observed in the control. Treated samples exhibited lower b* and C* values and higher h throughout the storage period.

**Table 5:**
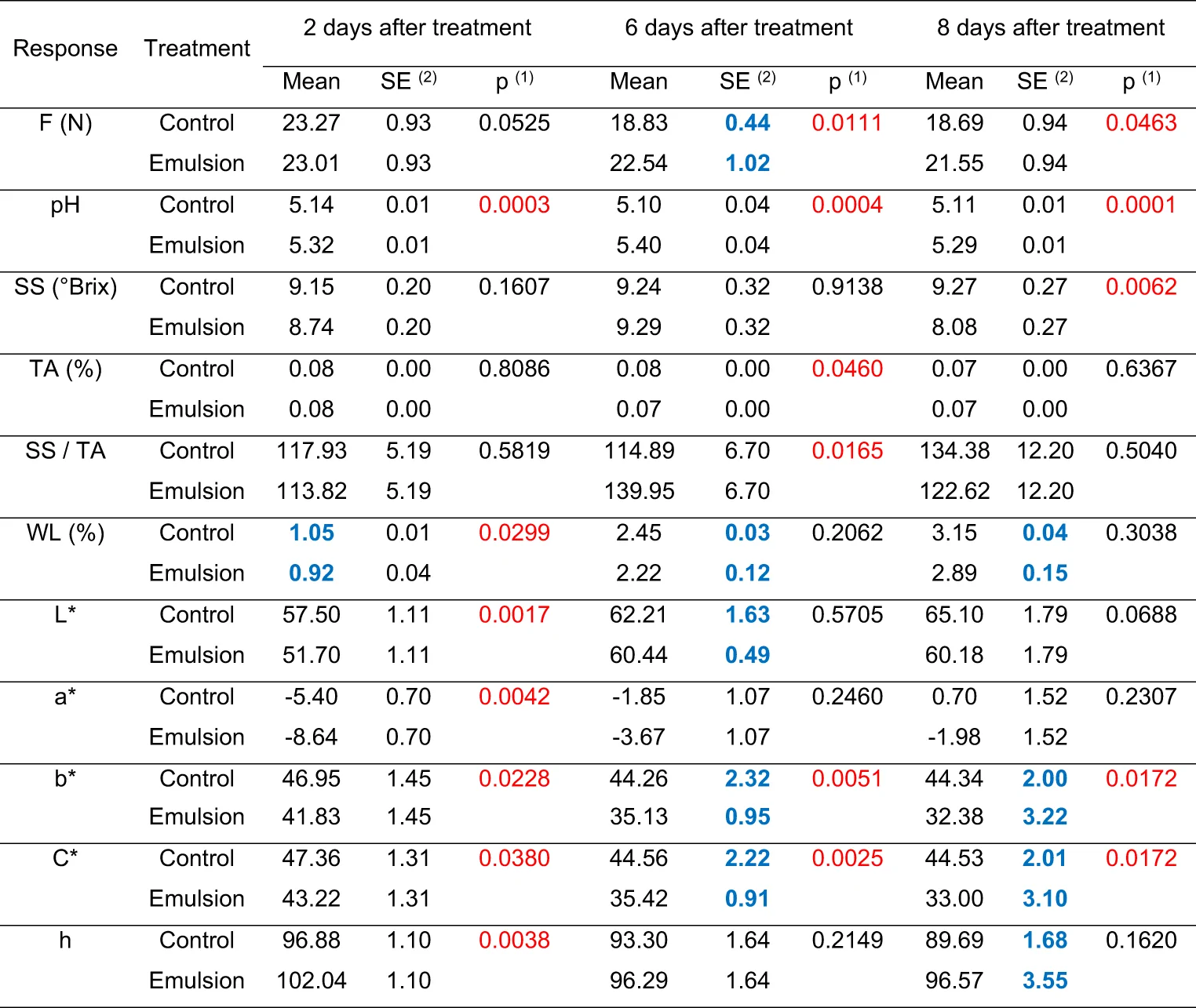
Estimated means (n = 10) of physicochemical parameters and their respective standard errors (SE) for untreated orange (control) or fruit treated with chitosan-based emulsion containing a synthetic mixture of carvacrol, thymol and p-cymene at final concentrations of 7.5 µL mL⁻¹. Firmness (F, Newton), pH, soluble solids (SS, °Brix), titratable acidity (TA, %), SS/TA ratio, weight loss (WL, %), and color (L*, a*, b*, C*, h)

^(1)^ Nominal significance level (p-value) of the Student’s t-test for independent samples. The p-values highlighted in red indicate significant differences between treatment means at the 0.05 significance level. ^(2)^ Standard errors (SE) were estimated from the analysis of variance (ANOVA) model. The values highlighted in blue correspond to comparisons for which Levene’s test indicated significant heterogeneity of variances (p < 0.05); therefore, the unequal-variance version of Student’s t-test was used for comparison of treatment means. Homogeneity of variances was assessed using Levene’s test, with the corresponding p-values reported in Supplementary Table S5

### Enzymatic analysis

**Fig. 3:**
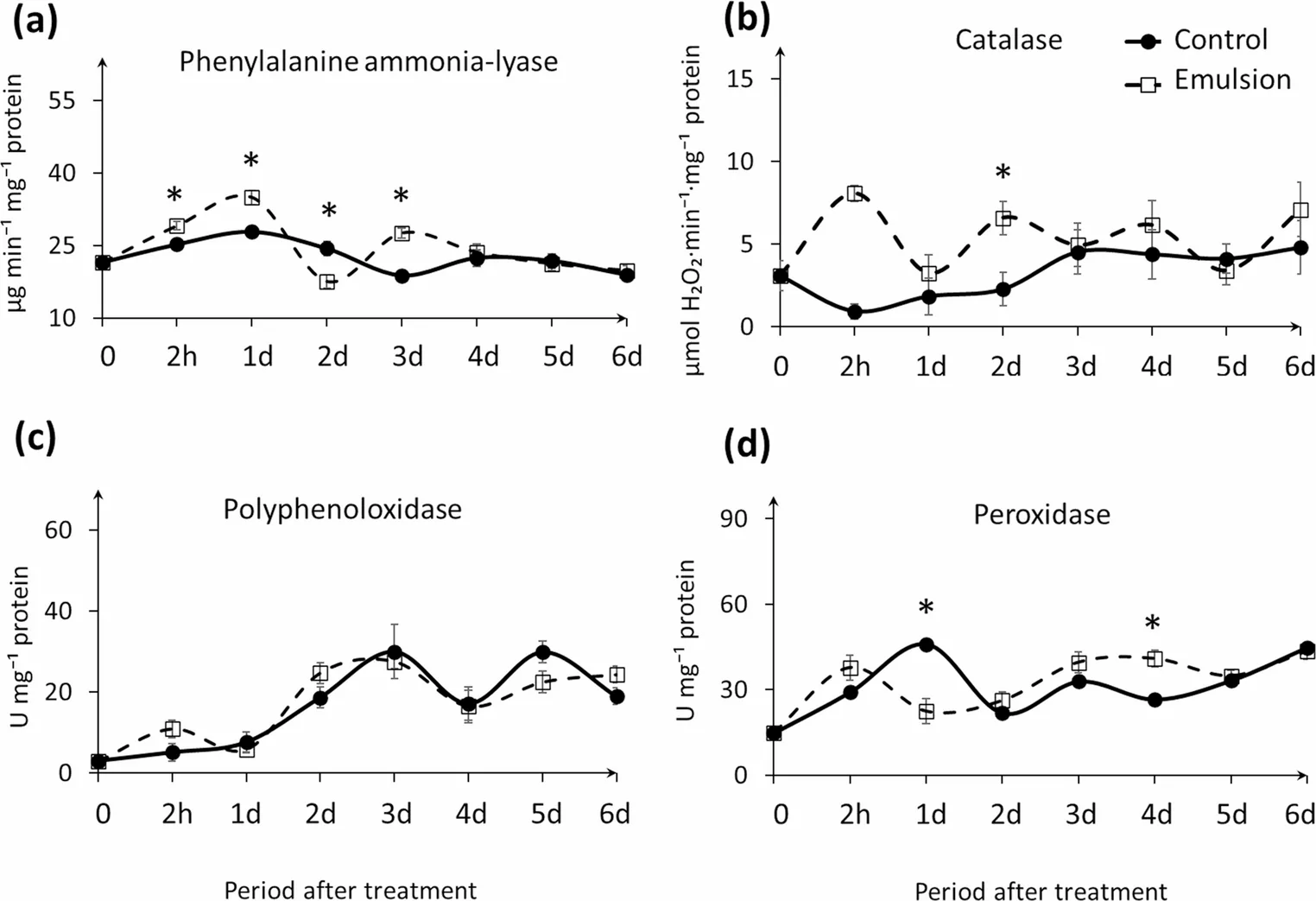
Activity of phenylalanine ammonia-lyase (**a**), catalase (**b**), polyphenol oxidase (**c**), and peroxidase (**d**) in orange fruit treated with chitosan-based emulsion containing a synthetic mixture of carvacrol, thymol, and p-cymene at a final concentration of 7.5 µL mL⁻¹. Enzyme activities were evaluated at 2 h and at 1, 2, 3, 4, 5, and 6 days after treatment. Means marked with an asterisk (*) indicate significant differences between the control and emulsion treatments according to Student’s t-test assuming equal variances; otherwise, the unequal-variance version of the test was applied. Vertical bars represent standard errors. Enzyme activities were expressed according to the specific assay used: phenylalanine ammonia-lyase as µg trans-cinnamic acid min⁻¹ mg⁻¹ protein; catalase as µmol H₂O₂ min⁻¹ mg⁻¹ protein; and polyphenol oxidase and peroxidase as U mg⁻¹ protein, where 1 U corresponds to an absorbance change of 0.01 min⁻¹

The application of the emulsion significantly affected the activity of defense-related enzymes at some of the evaluated sampling times (Fig. 3, Figure S5). Phenylalanine ammonia-lyase activity showed an increase during the first hours after treatment, with significantly higher values than the control at 2 h and on the first day. Lower values were observed on the second day, followed by higher values on the third day and a subsequent decline at later sampling times (Fig. 3a).

Catalase showed a similar pattern, with higher activity observed at 2 h after treatment, lower values on the first day, and a subsequent increase on the second day, when activity was significantly higher than in untreated fruit (Fig. 3b).

Polyphenoloxidase activity varied among the evaluated sampling times, although differences relative to the control were less consistent (Fig. 3c).

Peroxidase activity showed relatively small numerical changes among the evaluated sampling times, with significant increases on the first and fourth days compared with the control, followed by similar activity levels between treatments at subsequent sampling times (Fig. 3d).

## Discussion

In the direct-contact experiment in which the emulsion was poured over the spore suspension, complete inhibition of spore germination was achieved at 0.25 µL mL⁻¹. In the separate experiment in which the spore suspension was deposited over the emulsion-coated surface, a concentration of 3.0 µL mL⁻¹ resulted in complete inhibition of mycelial growth (Tables 1 and 2, Figure S2). The emulsion exhibited antifungal activity against *G. citri-aurantii* under both direct-contact experimental conditions. These findings demonstrate the antifungal activity of the formulation against *G. citri-aurantii* under the respective experimental conditions.

The antifungal activity observed in the present study may be related, at least in part, to the bioactive compounds present in the formulation. Carvacrol and thymol have been reported to interact with fungal cell membranes, increasing membrane permeability and causing leakage of intracellular components [43, 44]. These effects may compromise essential cellular processes and contribute to growth inhibition or cell death [43, 44]. However, these mechanisms were not directly evaluated in the present study, and the relative contribution of carvacrol and thymol to the antifungal activity of the complete formulation cannot be determined from the experimental design used here.

Although *p-cymene* has shown relatively low antifungal activity when evaluated alone [16], previous studies have suggested that it may interact with compounds such as carvacrol and thymol and facilitate their interaction with fungal cell [45]. Thus, the presence of p-cymene may have contributed to the antifungal activity observed in the present study. However, because synergism among the formulation components was not directly evaluated, this possibility should be considered a hypothesis based on the literature rather than a demonstrated synergistic effect.

The antifungal activity observed in the present study is consistent with recent evidence supporting the potential of oregano essential oil constituents and their combinations for the control of postharvest citrus pathogens. In a previous study, oregano essential oil and mixtures of its major constituents showed inhibitory activity against *G. citri-aurantii*, with minimum inhibitory concentrations ranging from 0.25 to 3.0 µL mL⁻¹ for the most active essential oils [16]. Notably, the mixture of the major constituents exhibited greater antifungal activity than the corresponding individual compounds, suggesting that interactions among constituents may contribute to the antifungal effect [16]. The present study extends these findings by demonstrating antifungal activity after incorporation of these compounds into a chitosan-based emulsion and by evaluating both direct-contact and postharvest applications.

Inhibition under volatile exposure was less pronounced than under direct-contact conditions. This difference indicates that the antifungal efficacy of the formulation varied according to the mode of exposure and may be influenced by the degree of contact between the formulation and the pathogen. Similar differences between direct-contact and vapor applications have been reported for thymol and carvacrol in the control of *Lasiodiplodia* spp., whose antifungal efficacy depends on their concentration in the surrounding atmosphere and exposure conditions [46]. Fungal cell membrane characteristics, including the presence of ergosterol, may also influence interactions with volatile compounds [47, 48]. However, because membrane properties and compound diffusion were not directly evaluated in the present study, these mechanisms remain possible explanations rather than experimentally demonstrated effects.

The in vivo experiment was conducted through direct application of the emulsion to the fruit surface, providing a practical postharvest application approach. The treatment at 7.5 µL mL⁻¹ showed the greatest numerical reduction in disease incidence and significantly reduced disease severity, as indicated by lower mean lesion diameter (MLD) and area under the disease severity progress curve (AUDPC) compared with untreated fruit. Although the 7.5 µL mL⁻¹ treatment also showed a numerically longer mean incubation period than the control, this difference was not statistically significant. These results indicate that the emulsion, particularly at 7.5 µL mL⁻¹, reduced the progression and severity of sour rot under the experimental conditions evaluated. Similar protective effects have been reported for citrus and other fruits treated with coatings containing essential-oil compounds. For example, an emulsion based on eugenol, carvacrol, and cinnamaldehyde reduced green mold development in mandarins [49], while chitosan coatings enriched with carvacrol suppressed fungal infections in guava [50]. Together, these findings reinforce the potential of essential-oil compound emulsions as alternative strategies for postharvest disease management.

The superficial damage observed on fruit treated with the highest emulsion concentration (10.0 µL mL⁻¹) represents an important limitation for practical application. Although this concentration showed antifungal activity, the occurrence of visible surface injury indicates that increasing the emulsion concentration does not necessarily result in a more suitable treatment. From a postharvest perspective, even slight surface damage may compromise marketability because visual appearance is an important quality attribute of fresh citrus fruit. Therefore, the results suggest that the concentration of 7.5 µL mL⁻¹ may provide a more favorable balance between disease control and fruit tolerance under the conditions evaluated in the present study. Nevertheless, this concentration should not yet be considered an optimal commercial dose, since further studies are required to establish the threshold between antifungal efficacy and fruit tolerance, particularly by evaluating a broader range of concentrations.

An additional limitation of the present study is the absence of sensory evaluation. Although the treatment at 7.5 µL mL⁻¹ provided disease control, the potential effects of the oregano-derived compounds on sensory attributes were not evaluated. Therefore, future studies should include sensory evaluation to determine whether the effective concentrations identified in the present study maintain consumer acceptability and to establish a more comprehensive relationship between antifungal efficacy, fruit quality, and sensory acceptance.

The enhanced antifungal efficacy observed in the present study may also be associated with the presence of *p-cymene* in the formulation. Previous studies have proposed that *p-cymene* may increase membrane fluidity and permeability, potentially facilitating the penetration of phenolic compounds such as carvacrol and thymol and contributing to their antifungal activity [51]. However, this proposed mechanism was not directly investigated in the present study and should therefore be considered a possible explanation for the observed antifungal effects.

Beyond the intrinsic antifungal activity of the bioactive compounds, the chitosan matrix may have contributed to the overall performance of the formulation. When applied to fruit surfaces, chitosan coatings can form semipermeable films that reduce water loss and modify gas exchange [50, 52]. Chitosan-based polymeric matrices have also been investigated as carrier systems for essential-oil constituents, with the potential to reduce volatilization and degradation of incorporated compounds [53]. These properties may have contributed to the performance of the formulation observed in the present study. However, because water vapor permeability, gas exchange, compound release, and pathogen penetration were not directly measured, these mechanisms cannot be confirmed from the present results.

However, because a formulation control containing the chitosan matrix without the oregano-derived bioactive compounds was not included in the present study, the individual contribution of the chitosan matrix cannot be distinguished from that of carvacrol, thymol, and p-cymene. Therefore, the antifungal and postharvest effects observed here should be interpreted as responses to the complete formulation rather than as effects attributable exclusively to the bioactive compounds. Future experiments including a chitosan-matrix control, as well as appropriate controls for the individual or combined bioactive compounds, would help clarify the contribution and possible synergistic effects of each component.

Although the chitosan emulsion exhibited a high polydispersity index (PDI = 1.0), indicating a highly heterogeneous droplet-size distribution, the stock emulsion remained physically stable, with no visible phase separation during at least one month of storage. The high polydispersity may be associated, at least in part, with the characteristics of the chitosan matrix, including its degree of deacetylation (80%), which may favor intermolecular interactions and aggregate formation [54]. Thus, the high PDI should be interpreted as a characteristic of the heterogeneous colloidal structure rather than as evidence of immediate macroscopic instability.

From a scalability and practical application perspective, some limitations of the formulation should also be considered. The emulsion was prepared and evaluated under laboratory-scale conditions, and therefore its physicochemical behavior, stability, and performance may differ during large-scale production. The broad droplet-size distribution observed in the present formulation may require optimization of the emulsification process and control of parameters such as mixing conditions, component proportions, and processing time to ensure batch-to-batch consistency. In addition, the stability of the formulation should be evaluated over longer storage periods and under different temperature conditions before large-scale application. Practical aspects such as compatibility with commercial postharvest application equipment should also be considered in future studies. Therefore, although the present results demonstrate the potential of the emulsion for postharvest disease management, pilot-scale studies are necessary to confirm formulation stability, reproducibility, application performance, and economic feasibility before commercial implementation.

For the antifungal assays, the 5% (v/v) stock emulsion was diluted with the same acidic chitosan solution to obtain the concentrations required for the experiments. Therefore, dilution increased the relative proportion of the chitosan matrix in the final formulations, which may contribute to modifying the colloidal organization and reducing the effective polydispersity. Nevertheless, this interpretation requires direct experimental verification and should not be considered a demonstrated mechanism in the present study.

The emulsion treatment also contributed to the preservation of physicochemical quality attributes during the experimental storage period. Treated fruit maintained higher firmness values than the control at the evaluated sampling times, indicating better retention of this quality attribute [55]. Maintenance of fruit firmness has been widely associated with the barrier properties of chitosan coatings, which reduce moisture loss and slow metabolic processes related to ripening [55].

Similarly, the higher pH values and the slight reduction in titratable acidity observed in treated fruit at the evaluated sampling times may be associated with differences in organic acid metabolism between treatments. This response may also be related to the barrier properties of chitosan coatings, which have been associated with modifications in gas exchange and respiration in coated fruits [56, 57]. However, respiration rates were not directly measured in the present study. Bioactive compounds such as carvacrol and thymol have also been reported to influence oxidative metabolism and reactive oxygen species (ROS), which may contribute to the maintenance of fruit quality [58]. These mechanisms therefore represent possible explanations based on previous literature rather than effects directly demonstrated in the present study.

The lower weight loss observed in treated fruit at the evaluated sampling times further supports the protective effect of the coating. Chitosan films are known to reduce water vapor diffusion and limit physiological deterioration [56, 57].

Changes in peel color at the evaluated sampling times reflected the natural ripening process of oranges, characterized by increases in L* and a* values. However, fruit treated with the emulsion exhibited lower C* values and more stable h* values compared with the control. These results indicate that the coating contributed to maintaining more stable peel coloration under the experimental conditions. Bioactive compounds present in the formulation possess antioxidant properties that may reduce oxidative degradation of pigments, thereby helping to preserve visual quality [59].

In addition to its direct antifungal effects, the emulsion was associated with changes in the activity of enzymes related to plant defense, suggesting that the treatment may also stimulate defense-related responses in the fruit. The higher phenylalanine ammonia-lyase activity observed after treatment at specific sampling times may be consistent with modulation of the phenylpropanoid pathway, which is involved in the synthesis of phenolic compounds that contribute to plant defense and may possess antimicrobial and antioxidant properties [60, 61].

Catalase activity also showed higher values after treatment at specific sampling times. Because catalase is involved in the removal of hydrogen peroxide (H₂O₂), this response may indicate an increased capacity of the fruit tissue to regulate reactive oxygen species (ROS) following treatment. The coordinated regulation of ROS is an important component of plant responses to biotic and abiotic stimuli and may contribute to the maintenance of cellular redox balance [60].

Polyphenoloxidase showed a variable response among the evaluated sampling times. This pattern may reflect differences in the availability of phenolic substrates and in the physiological status of the fruit at the respective sampling times. Polyphenoloxidase is involved in the oxidation of phenolic compounds and may contribute to the formation of quinones and subsequent polymerization reactions associated with defense and tissue strengthening [62].

Peroxidase activity showed relatively small numerical differences among the evaluated sampling times, suggesting that this enzyme was not substantially modulated by the treatment under the conditions evaluated [63].

Overall, the antifungal and postharvest effects observed in this study are supported by the results obtained in our experiments. However, the mechanisms proposed in the Discussion are mainly based on previous studies and were not directly evaluated here. Respiration, ROS accumulation, membrane integrity, cell-wall degradation, compound release, pathogen colonization, and interactions among the formulation components were not measured. Therefore, these mechanisms should be considered possible explanations rather than confirmed mechanisms. Further studies are needed to confirm how the formulation produces the effects observed in this study.

## Conclusion

The chitosan emulsion containing the major constituents of oregano essential oil (carvacrol, thymol, and *p-cymene*) displayed in vitro antifungal activity against *G. citri-aurantii*, with complete inhibition of colony formation at 0.25 µL mL⁻¹ and total suppression of mycelial growth from 3 µL mL⁻¹ onward. Although the volatile phase was less effective than direct contact, it still exerted a noticeable inhibitory effect at a concentration of 50 µL mL⁻¹.

The emulsion at 7.5 µL mL⁻¹ reduced sour rot severity and was associated with better retention of physicochemical and color attributes under the experimental conditions. The treatment was also associated with changes in the activity of selected defense-related enzymes. However, these responses should be interpreted as modulation of defense-related biochemical processes rather than as definitive evidence of induced resistance.

Overall, the results indicate that the chitosan emulsion is a promising alternative approach for postharvest management of sour rot in ‘Lima’ orange. However, further studies are needed to determine the optimal application concentration, assess sensory acceptance, evaluate formulation stability and performance under pilot- and large-scale conditions, and clarify the mechanisms underlying the observed enzymatic responses before its practical or commercial application can be established. 

## Supplementary Information

Below is the link to the electronic supplementary material.

Supplementary Material 1

## Data Availability

The authors declare that the data generated in this study will be available upon request.
