# Supplementary material for: Chitosan-based edible coating produced from an emulsion containing the major constituents of oregano essential oil for the control of sour rot in ‘Lima’ orange
Source: Braz J Microbiol. 2026 Oct 23;57(1):304. doi: 10.1007/s42770-026-02125-1 (PMC13694614; doi:10.1007/s42770-026-02125-1)
Supplement: Supplementary file 1 [file 42770_2026_2125_MOESM1_ESM.docx]

**Supplementary material**

**Table S1.** Characteristics of the major constituents of oregano essential oil (*Origanum vulgare*) and their respective mass fraction used in the formulation of the synthetic mixture.

| Constituents ^(1)^ | Density  (g cm^-3^) | Volume (mL) | Mass (g) | Mass (%) | n (mmol) |
| --- | --- | --- | --- | --- | --- |
| Carvacrol | 0.976 | 3.76 | 3.67 | 77.20 | 0.024 |
| *p-cymene* | 0.857 | 1.02 | 0.88 | 18.50 | 0.006 |
| Thymol | 0.965 | 0.21 | 0.20 | 4.30 | 0.001 |
| ^(1)^ All reagents were purchased from Sigma-Aldrich (St. Louis, Missouri, USA). | | | | | |

**Figure S1.** Overlap of the optical and Confocal fluorescence images of the emulsion. The red spots represent the droplets of the major components of the oregano essential oil. The dark regions represent the chitosan solution matrix and some air bubbles. The inset summarizes the major physicochemical parameters obtained by dynamic light scattering (DLS) and electrophoretic mobility measurements, including Z-average hydrodynamic diameter, polydispersity index (PDI), and zeta potential. The chemical structures and relative proportions of the oregano oil constituents used to prepare the synthetic mixture are shown on the right: carvacrol (77.2%), *p-cymene* (18.5%), and thymol (4.3%). Scale bar: 50 μm.

**Figure S2.** Effect of the antifungal activity of applying an emulsion produced with chitosan solution containing the major constituents of oregano (*Origanum vulgare*) essential oil - carvacrol, thymol, and *p-cymene* on the control of *Geotrichum citri-aurantii*, isolated from orange fruit, in different *in vitro* experiments: a) Indirect contact inhibition: volatile phase, b) Direct contact inhibition by depositing the emulsion on the spore suspension, c) Direct contact inhibition by depositing a paper disc with the spore suspension on the emulsion.

**Figure S3.** Orange fruit variety ‘Lima’ treated with an emulsion produced with chitosan solution containing the major constituents of oregano (*Origanum vulgare*) essential oil - carvacrol, thymol, and *p-cymene*, at concentrations of 2.5, 5.0, 7.5, and 10.0 µL mL⁻¹, before and after 6 days of storage.

**Table S2.** User-defined contrast matrix used for nonparametric multiple comparisons of emulsion concentrations in the direct-contact *in vitro* experiments.

| Contrast | 0.10 µL mL⁻¹ | 0.25 µL mL⁻¹ | 0.50 µL mL⁻¹ | Direction tested |
| --- | --- | --- | --- | --- |
| C1 | 1 | −1 | 0 | 0.25 > 0.10 |
| C2 | 1 | 0 | −1 | 0.50 > 0.10 |
| C3 | 0 | 1 | −1 | 0.50 > 0.25 |
| The one-sided alternative hypothesis was specified as alternative = "*less*". Under the parameterization used by *nparcomp*, the contrasts were oriented such that the second group was expected to have a greater response than the first. | | | | |

**Table S3.** User-defined contrast matrix used for nonparametric multiple comparisons of emulsion concentrations in the direct-contact *in vitro* experiments.

| Contrast | 2.00 µL mL⁻¹ | 3.00 µL mL⁻¹ | 4.00 µL mL⁻¹ | Direction tested |
| --- | --- | --- | --- | --- |
| C1 | 1 | −1 | 0 | 3.00 > 2.00 |
| C2 | 1 | 0 | −1 | 4.00 > 2.00 |
| C3 | 0 | 1 | −1 | 4.00 > 3.00 |
| The one-sided alternative hypothesis was specified as alternative = "*less*". Under the parameterization used by *nparcomp*, the contrasts were oriented such that the second group was expected to have a greater response than the first. | | | | |

**Table S4.** Dunnett contrast matrix used for nonparametric multiple comparisons of emulsion treatments with the untreated control in the *in vivo* experiment.

| Contrast | 0.00 µL mL⁻¹ | 2.50 µL mL⁻¹ | 5.00 µL mL⁻¹ | 7.50 µL mL⁻¹ | 10.00 µL mL⁻¹ |
| --- | --- | --- | --- | --- | --- |
| 2.50 – 0.00 | -1 | 1 | 0 | 0 | 0 |
| 5.00 – 0.00 | -1 | 0 | 1 | 0 | 0 |
| 7.50 – 0.00 | -1 | 0 | 0 | 1 | 0 |
| 10.00 – 0.00 | -1 | 0 | 0 | 0 | 1 |
| The Dunnett contrast matrix was automatically generated by nparcomp using type = "Dunnett". The same contrast matrix was used for all in vivo response variables, with the one-sided alternative hypothesis specified according to the expected direction of the treatment effect. For MLD and AUDPC, alternative = "less" was used to test whether the response in each emulsion treatment was lower than that in the untreated control. For the incubation period, alternative = "greater" was used to test whether the response in each emulsion treatment was greater than that in the untreated control. | | | | | |

**Table S5.** P-values from Levene’s test for homogeneity of variances for physicochemical variables evaluated in untreated orange fruit (control) and fruit treated with a chitosan emulsion containing a synthetic mixture of carvacrol, thymol, and p-cymene at 7.5 µL mL⁻¹, at 2, 6, and 8 days after treatment. Firmness (F, Newton), pH, soluble solids (SS, °Brix), titratable acidity (TA, %), SS/TA ratio, weight loss (WL, %), and color (L*, a*, b*, C*, h).

| Response | 2 days after treatment | 6 days after treatment | 8 days after treatment |
| --- | --- | --- | --- |
| F (N) | 0.2840 | 0.0173 | 0.4787 |
| pH | 0.3399 | 0.4523 | 1.000 |
| SS (°Brix) | 0.5815 | 0.4950 | 0.7353 |
| TA (%) | 0.7141 | 0.1964 | 0.0987 |
| SS / TA | 0.8985 | 0.1701 | 0.0796 |
| WL (%) | 0.0065 | 0.0066 | 0.0058 |
| L* | 0.2669 | 0.0015 | 0.0655 |
| a* | 0.8981 | 0.0503 | 0.3088 |
| b* | 0.2238 | 0.0017 | 0.0318 |
| C* | 0.3286 | 0.0012 | 0.0456 |
| h | 0.6203 | 0.0921 | 0.0073 |
| P-values correspond to Levene’s test for homogeneity of variances between the control and emulsion treatments at each evaluation time. A p-value < 0.05 indicates rejection of the null hypothesis of equal variances. For comparisons with p < 0.05, treatment means were compared using the unequal-variance version of Student’s t-test (Welch’s t-test). Standard errors (SE) reported in Table 4 were estimated from the analysis of variance (ANOVA) model and are presented independently of the Levene’s test results. | | | |

**Figure S4.** Orange fruit variety ‘Lima’ treated with an emulsion produced with chitosan solution containing the major constituents of oregano (*Origanum vulgare*) essential oil - carvacrol, thymol, and *p-cymene*, at a concentration of 7.5 µL mL⁻¹, intended for the evaluation of physicochemical parameters upon arrival of the fruit at the laboratory and after 2, 6, and 8 days of storage.

**Figure S5.** Orange fruit variety ‘Lima’ treated with an emulsion produced with chitosan solution containing the major constituents of oregano (*Origanum vulgare*) essential oil - carvacrol, thymol, and *p-cymene*, at a concentration of 7.5 µL mL⁻¹, were used in enzymatic experiment. The images were taken immediately after the treatment application and do not correspond to the day of the enzymatic evaluation.
